# First detection of *Gongylonema* species in *Geotrupes mutator* in Europe

**DOI:** 10.21307/jofnem-2021-050

**Published:** 2021-05-21

**Authors:** Daniel Bravo-Barriga, Manuel Martín-Pérez, Jorge M. Lobo, Ricardo Parreira, Juan Enrique Pérez-Martín, Eva Frontera

**Affiliations:** 1 Parasitology Area, Department of Animal Health, Veterinary Faculty, University of Extremadura, Cáceres, Spain; 2 Department of Biogeography and Global Change, Museo Nacional de Ciencias Naturales, CSIC, Madrid, Spain; 3 Global Health and Tropical Medicine (GHTM), Instituto de Higiene e Medicina Tropical (IHMT), Universidade Nova de Lisboa (UNL), Grupo de Virologia/Unidade de Microbiología Médica, Lisbon, Portugal

**Keywords:** Beetles, *Geotrupes*, *Gongylonema*, Host-parasitic relationship, Molecular biology, Nematodes, Spain

## Abstract

The detection of three *Gongylonema* sp. infective larvae in two specimens of the dung beetle *Geotrupes mutator* (Marsham, 1802) from western Spain is reported here for the first time in Europe. Scanning electron microscopy confirmed that the analyzed specimens belong to the genus *Gongylonema*, but it was not possible to determine the species identity by the lack of morphological information in the literature and because many of the phenotypic characteristics had not yet fully developed at this juvenile stage. Nevertheless, a phylogenetic analysis using amplified *cox1* nucleotide sequences has revealed that the studied larvae could be clearly discriminated (< 89% identity) from all the other *Gongylonema cox1* sequences available in public genetic databases. While our results are limited by the scarcity of genetic information available for this genus, the possibility that the analyzed specimens might correspond to a new species should not be ruled out, and more studies are needed. The results provided in this report indicate that *G. mutator* is involved in the transmission cycle of *Gongylonema* sp. to vertebrates in Europe.

Under the functional name of “dung beetles”, approximately 9,500 worldwide species have been grouped into the Aphodiinae, Scarabaeinae, and Geotrupinae subfamilies of the superfamily Scarabaeoidea (Löbl and Smetana, 2016). Dung beetles are considered the most important agents promoting the recycling of mammal faeces in terrestrial ecosystems. They contribute to the nitrification and aeration of the soil, seed dispersal, and the control of fly and cattle parasites (Nichols et al., 2008). However, as dung beetle species are in close contact with animal and human faeces, they may also act as hosts for a wide variety of pathogenic bacteria and/or viruses, as well as eukaryotic parasites favouring their dispersion and transmission (Nichols et al., 2017). This is particularly true for different platyhelminthes (flatworms) and nematode (roundworms) belonging to the genera *Gongylonema*, *Spirocerca*, *Ascarops*, *Acanthocephalus*, *Macracanthorynchus*, or *Physocephalus* (Poinar, 1975). These parasites use dung beetles as intermediate, incidental, or paratenic hosts (Mowlavi et al., 2009; Mukaratirwa et al., 2010; Nichols and Gómez, 2014) affecting numerous species of birds, other domestic or wild mammals, as well as humans (Kinsella et al., 2016; Mukaratirwa et al., 2010; Nichols and Gómez, 2014).

*Gongylonema* Molin, 1857 (Spirurida: Gongylonematidae) is a genus that includes approximately 50 species, which are known parasites of birds and mammals, including humans (Cordeiro et al., 2018; Kinsella et al., 2016). However, due to variations of critical phenotypic characters, morphological identification of these species is difficult and their number may have been underestimated (Setsuda et al., 2018). *Gongylonema* infection is a neglected zoonotic parasitic disease, reported in several countries, that is mainly caused by consumption of contaminated water, raw food, or accidental ingestion of host insects (Xiaodan et al., 2018). *Gongylonema* species follow a terrestrial indirect life cycle involving approximately 50 species of insects as intermediate hosts, such as dung beetles and cockroaches (Mowlavi et al., 2009; Mukaratirwa et al., 2010). However, the paucity of available observations prevents estimating the network of relationships between these parasites and dung beetles (Nichols et al., 2017). In the present study, we aim to improve the knowledge about these relationships by describing, for the first time, a *Gongylonema* species hosted by a European Geotrupinae species, and also comparing the genetic relationships of the specimens found with other nematode taxa by analyzing the mitochondrial *cox1* genetic sequences.

## Materials and methods

In October 2017, a sampling of beetles captured by 27 pitfall traps was carried out according to a standardized sampling procedure (Lobo et al., 1988). The collections were carried out in three localities of western Spain, in the provinces of Salamanca and Zamora. Three types of excrements (pig, bovine, canine) were used as bait or attractant at each location. The captures of each trap were collected 65−80 h after trap placements and immediately deposited in 10 × 10 × 10 cm plastic containers containing soil and stored at 15°C during transport to the laboratory. The collected adult specimens with infective larvae were identified using morphological identification keys provided by Martín-Piera and López-Colón (2000). The beetles were dissected under a stereoscope for the detection of nematode larvae. In case of infection, larvae were placed in 70% ethanol until molecular analyses could be performed, with the exception of one worm selected for optical (× 100−200; Nikon eclipse 80i and digital photomicrography system, DXM1200F) and scanning electron (SEM) microscopy. For SEM, specimens were dehydrated through a graded series of ethanol from 70 to 100%, freeze-dried with liquid CO_2_ according to the critical point method, and mounted onto stubs. Mounting of the sample on the sample holder was done using double-sided carbon adhesive tape. Mounted specimens were then sputter-coated with a gold layer (20 nm ± 5%) and observed with an FE-SEM Hitachi S-4800 (FE by Field Emission, with Field Emission Electron Gun).

Genomic DNA (gDNA) was extracted from individual worms found in beetles using an NZY tissue gDNA kit (NZYTech) following the manufacturer’s instructions. The *cox1* genes were partially amplified using the primer set NTF and NTR following a PCR protocol previously described for the analysis of Spirurida (Casiraghi et al., 2001), and the product was sent for sequencing to STABvida (https://www.stabvida.com/es). Sequences were edited in Chromas Lite 2.1.1 (Technelysium Pty Ltd) and consensus sequences for each forward/reverse pair were created in BioEdit Sequence Alignment Editor (version 7.2.5, Carlsbad, CA. USA). Finally, the nucleotide sequences obtained (> 652 pb in length) were deposited at the DNA Data Bank of Japan (DDBJ: accession numbers LC577505 and LC612845). In addition to the specimens collected in the course of this study, a DNA extract prepared using a formerly unidentified *Gongylonema* specimen, obtained from *Otus scops*, Spain, and analyzed in a previous study (Esperón et al., 2013), was provided by its authors in order to obtain its corresponding sequence for the *cox1* barcode section (DDBJ: accession numbers LC620542).

Phylogenetic analyses were carried out based on the construction of multiple alignments of nucleotide (nt) sequences either comprising a wide range of nematodes (including *Cylicospirura, Dirofilaria*, *Gongylonema*, *Mastophorus, Onchocerca, Physaloptera, Setaria*, *Spirocerca*, and *Thelazia*; *n* = 31) or restricted to the *Gongylonema* genus (*n* = 18). These were constructed using the iterative G-INS-I method as implemented in MAFFT vs. 7 (https://mafft.cbrc.jp/alignment/server/) followed by their edition using GBlocks (http://molevol.cmima.csic.es/castresana/Gblocks_server.html). Phylogenetic trees were built using the Maximum Likelihood (ML) optimization criterium and the Mega X software (Kumar et al., 2018), and their topological stability was assessed by bootstrapping with 1,000 re-samplings of the aligned sequence data. Alternatively, a Bayesian approach was also used for phylogenetic reconstruction, taking advantage of the BEASTv1.10.4 software (Suchard et al., 2018). For both situations, the best-fitting evolutionary model used was GTR + Γ+ I. Bayesian analyses included two independent Markov chain Monte-Carlo (MCMC) runs carried out assuming a lognormal-relaxed molecular clock (as suggested by Mega X Molecular Clock test), and a constant population coalescent prior. These runs were executed until 1 × 10^8^ states were sampled (10% of which were discarded as burn-in), and the final tree sample was summarized as a maximum clade credibility (MCC) tree.

Intra- and inter-group genetic distances were calculated using the Tamura/Nei model with gamma-distributed rates (*α* = 0.65), as suggested by Mega X. Sequence similarity searches were carried out using BLASTn (MegaBlast option; https://blast.ncbi.nlm.nih.gov/Blast.cgi) and Barcode of Life Data Sytems-v4 (http://www.boldsystems.org/). Finally, a principal coordinate analysis was carried out using PCOORD (http://www.hiv.lanl.gov/content/sequence/PCOORD/PCOORD.html)to visualize the genetic relationships between the *cox1* sequences of the different *Gongylonema* species.

## Results and discussion

A total of 551 dung beetle specimens were analyzed and three nematode infective larvae were detected in two specimens of *Geotrupes mutator* (Marsham, 1802), indicating a prevalence of 0.36% over the total of beetle specimens sampled. One of the beetles hosted a single larva, the other dung beetle specimen hosted two *Gongylonema* larvae. They were captured in Campillo de Azaba (Salamanca province; Lat: 40.510, Long: –6.689), in a valley area and a contiguous holm oak forest area, respectively. Both specimens were collected in pitfall traps baited with pig dung.

Members of the genus *Gongylonema* are easily recognized because their cuticle is covered by large verruciform thickenings, especially prominent on the anterior part of the body. By optical microscope, the morphological features of one specimen clearly indicated the presence of a third larval stage of *Gongylonema*. The total length of the specimen was 1.55 mm, width at mid-body of 0.075 mm, and a distance from the anus to the posterior end of the body of 0.085 mm (Fig. 1.1). The cephalic structure showed the typical mouthparts of this genus (Fig. 1.2).

**Figure 1:**
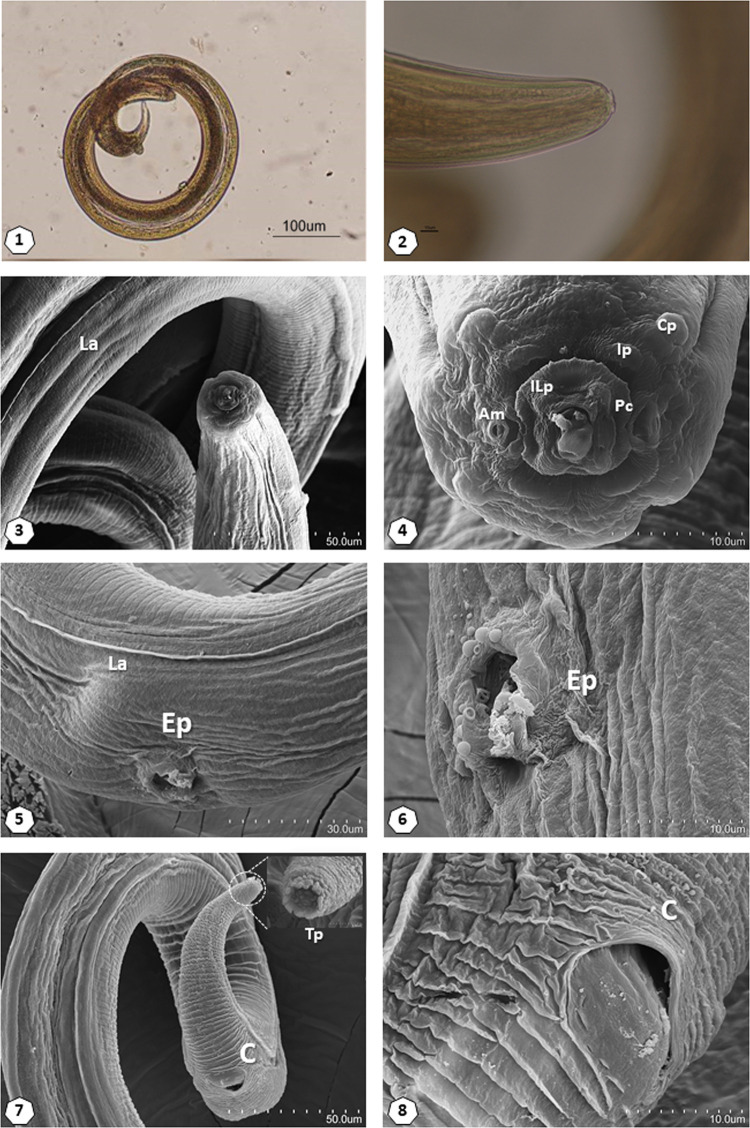
Scanning electron microscopy (SEM) of *Gongylonema* sp. (1) Anterior portion of a specimen L3 without cuticular bosses and highlighting the lateral ala (La). (2) Central view of the cephalic end, highlighting the amphids (Am), 4 cervical papillae (Cp), 4 internal papillae (Ip), 6 inner lateral papilla (ILp), peribuccal collar (Pc) and cuticular plates on the margins of the buccal opening. (3) Detail of lateral alae (La) and excretory pore (Ep). (4) Details of excretory pore (Ep). (5) Posterior portion of a specimen L3 with a view of the cloacal opening (C) and details of caudal end (Tp). (6) Details of Cloaca (C).

The detailed observation by SEM showed the classic elevation peri buccal collar present in its internal face (Fig. 1.4), as well as an inner circle of labial papillae constituted by six well-developed labial papillae arranged in two groups of three papillae located laterally. In the external face of the peri buccal collar, there was an outer circle of labial papillae formed by four small papillae (Fig. 1.4). More externally, a circle of cephalic papillae constituted by four large papillae could also be observed (Fig. 1.5). Two large amphids were located lateral to the mouth and two lateral depressions. Two lateral alae extend throughout the body length (Fig. 1.6). Finally, the caudal region of the worm was adorned with a tuft of a dozen cuticular fingerings (Fig. 1.7).

Regarding molecular analyses, two partials nematode *cox1* sequences were obtained (accession numbers LC577505 and LC612845) from specimens collected in the course of this study. The data coming from an additional specimen previously described from Madrid (Esperón et al., 2013) (accession numbers LC620542) was also included in the genetic analyses performed in this study. A search for similar sequences using Blastn unambiguously identified (E values = 0) several *cox1* homologues among *Gongylonema* nematodes, indicating high similarity with *G. nepalensis* and *G. pulchrum*. However, while the matches covered > 97% of our sequences, the identity percentage for the best-fit ones averaged only 88%. On the other hand, a Blast-2seq analysis revealed that the *cox1* LC620542 sequence (from Madrid) shared < 87% identity with both LC577505 and LC612845. Moreover, an attempted automatic molecular identification of the Spanish sequences in question using the BOLDSYSTEMS *cox1*-based molecular identification engine failed to produce any best match.

**Figure 2:**
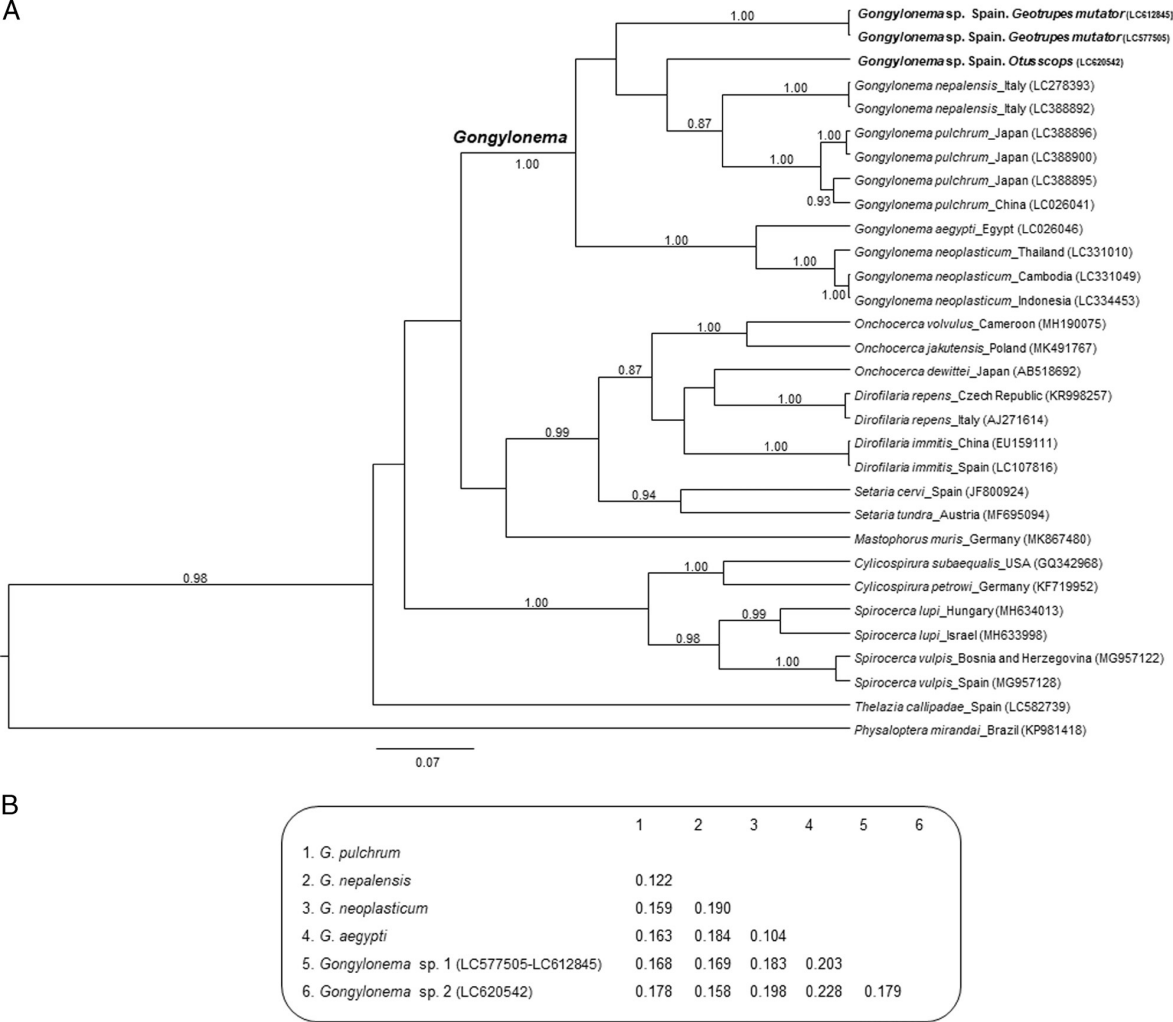
(A) Phylogenetic analysis (MCC tree obtained using a Bayesian approach) of *cox1* sequences (*n* = 47) from 10 different genera of nematodes. At specific branches, the values indicate the topological branch-support, as revealed by posterior probability values > 0.80. The branch defining the *Gongylonema* genus is indicated. The size bar indicates the number of nucleotide substitutions per site. The *cox1* sequences obtained in the course of this study are highlighted in bold case. (B) Estimates of evolutionary divergence of *cox1* over sequence pairs between groups. The analysis involved 16 *Gongylonema* sp. sequences.

The Bayesian phylogenetic tree including many genera of nematodes (*Cylicospirura*, *Dirofilaria*, *Gongylonema*, *Mastophorus*, *Onchocerca*, *Physaloptera*, *Setaria*, *Spirocerca,* and *Thelazia*) (Fig. 2a) indicated that the *cox1* sequences of the collected specimens were associated with those corresponding with to the *Gongylonema* genus. Furthermore, the collected specimens unambiguously segregated away from all the monophyletic clusters that defined the different species in the *Gongylonema* radiation (*G. aegypti*, *G. neoplasticum*, *G. nepalensis*, and *G. pulchrum*) (Fig. 2a). Interestingly, the two specimens hosted by *G. mutator* are grouped together, but the third one obtained from *Otus scops* in Madrid was segregated in a sister branch, suggesting that we could be dealing with two different *Gongylonema* species.

**Figure S1:**
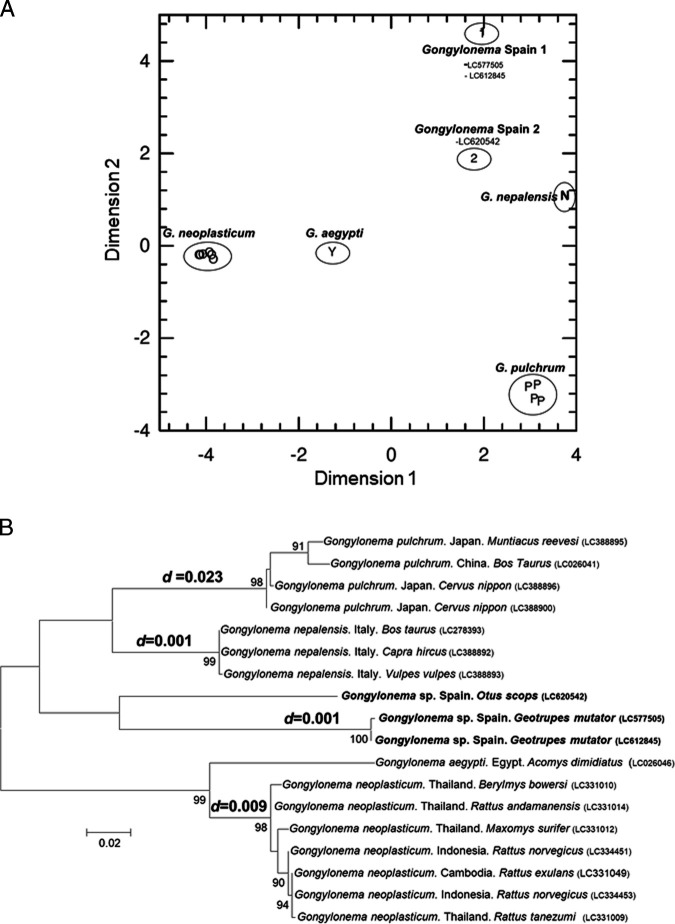
Supplementary document: (A) Multivariate principal coordinate analysis (PCOORD) for the dataset of sequences analysed in Maximum Likelihood tree, and that include *G. aegypti*, *G. neoplasticum*, *G. nepalensis*, and *G. pulchrum*, indicated by Y, O, N and P, respectively (used as references). The sequences obtained in the course of this study are indicated as Spain 1 and 2. The axes of this graph correspond to the two dimensions that were first extracted; together they cover over 66% of the total differences between the groups identified. (B) Phylogenetic analysis (Maximum Likelihood tree) of *cox1* sequences (*n* = 18) amplified from *Gongylonema* nematodes. At specific branches, the values indicate the branch-support as revealed by bootstrap analysis, with values ≥ 75 defining high topological support. The size bar indicates the number of nucleotide substitutions per site. The *d* values above the *G. neoplasticum*, *G. nepalensis* and *G. pulchrum* indicate intra-species genetic distances.

The analysis of the genetic distances supported the suggestion that the three considered specimens belong to 2 different *Gongylomena* species since the genetic distances between them are similar to those shared by the other reference species (*G. aegypti*, *G. neoplasticum*, *G. nepalensis*, and *G. pulchrum*) (Fig. 2b). This separation was further confirmed by the Principal Coordinate Analysis (PCOORD) (Fig. S1A). Finally, as expected, when a phylogenetic tree only including *Gongylomena cox1* sequences was obtained (Fig. S1B), the sequences here reported branched away from all the other species-defining branches. Although we cannot confirm the specific taxonomic status of the collected *Gongylonema* specimens, the analysis of the obtained *cox1* sequences improve our understanding of the interspecific genetic divergences of this little-studied genus.

In Spain, seven species of genus *Gongylonema* have been reported (Cordero et al., 1994; Fernandez-De-Mera et al., 2003; da Costa Cordeiro et al., 2018). Although we have not been able to find out which specie is our specimens, must a priori rule out *G. pulchrum* and *G. neoplasticum* present in Spain based on genetic analysis. Knowing that adults of *Gongylonema* (*Progongylonema*) are distinguished mainly from those of the subgenera *Gongylonema* (*Gongylonema*) and *Gongylonema* (*Gongylonemoides*) by the absence of cuticular bosses, their inclusion in this subgenus would be probable since this morphological character has not been observed in the specimen here analyzed. However, it is possible that our specimen may not yet have all the developed characteristics, as other authors observed for the different stages in other species (Alicata, 1935). The only known member of this subgenus in Spain is *G.* (*Progongylonema*) *pacoi* which was described for the first time in 1992 parasitizing the mucosa under the tongue of Corvids from the north of Cordoba province (Southern Spain) (Hernandez-Rodriguez and Gutiérrez-Palomino, 1992). Another *Gongylonema* that was detected in birds in Spain, was found in Madrid in various specimens of *Otus scops* (Esperón et al., 2013). Although the taxonomic identity of this species could not be established initially, our results have suggested the do not correspond to any of the *Gongylonema* species with available public genetic data. This would support the existence of a higher diversity of this genus in Spain. On the other hand, the infesting larva of *G. soricis* is similar to our nematode due to its close size (1545 µm-1580 µm) and its caudal ornamentation adorned with a tuft of cuticular fingerings (Quentin and Gunn, 1981). *G. soricis* has been rarely collected in Spain, except for records in Valencia in 1996 (Portoles et al., 1996). Taking into account these data and that its definitive host is grey shrew (*Crocidura russula*) which are highly distributed in Spain, *G. soricis* could be a good candidate for the identity of our specimen analysed in this study. Other potential candidate species include *G. (Gongylonematidae) pithyusensis* n. sp. and *G. mucronatum*. The first was described for the first time in 1977 parasitizing the oesophagus mucosa of the dormouse *Eliomys quercinus* (Mas Coma, 1977) in the Balearic Islands, Spain. Since then, it has only been detected once in Murcia (southeast) (Esteban et al., 2004), while the second was reported from *Atelerix algirus* (North African hedgehog) in North Africa and Spain (on the Balearic islands and Valencia: see Ferrer Màrius Vicent et al. (2000). However, as the distribution of *A. algirus* is limited to the east of the Iberian Peninsula, it is unlikely that our specimens belong to this species. The last species of *Gongylonema* inhabiting the Iberian peninsula is *G. musculi*, which had been recorded occasionally on several Mediterranean islands (Milazzo et al., 2003). The limited information about the morphology of the infecting larvae of this species prevent us from making any comparison. Therefore, the lack of morphological and genetic information on Iberian *Gongylonema* species momentarily makes a more specific definitive determination impossible.

While knowledge on the presence of nematodes in beetles from Spain could be regarded as helpful, it is either still scarce, or in the case of *Gongylonema*, non-existent. Although species of the genera *Ascarops, Diplocapter, Diplogaster, Eudiplogaster, Holodiplogaster, Physocephalus, Pelodera, Paroigolaimella, Rhabditis,* and *Spirocerca* have been detected in *G. mutator*, only *G. pulchrum* had been reported (Poinar, 1975). Therefore, this is the first report of other *Gongylonema* sp. in *G. mutator* naturally infected, at least in Europe. *G. mutator* is a widely distributed species in Europe from the south of Sweden to the Caucasus and the Iberian Peninsula, that also inhabit some Asian regions of Iran Kazakhstan, and Turkey (Löbl and Smetana, 2016). In the Iberian Peninsula this dung beetle species is frequent in the northern half part (Martín-Piera and López-Colón, 2000).

The only infected beetles with the analysed larvae were collected in traps baited with pig excrements. In general, Geotrupinae species are frequently associated with dung of mammalian herbivores (Martín-Piera and López-Colón, 2000). *G. mutator* digs several tunnels immediately below, or to the side, of the cow and horse dung pats (Teichert, 1955), using them for feeding or nesting. Cows and horses are thus the most probable candidates to host this parasite, although the general low trophic specificity of dung beetles (Martin-Piera and Lobo, 1996) has not yet made this point any clearer.

According to the morphologic and genetic analysis on the taxonomic identity of the *Gongylonema* species from *G. mutator*, there are the following possible interpretations:

- The lack of genetic data of other *Gongylonema* species makes it impossible to relate this *Gongylonema* species with another already described only using morphological characters of adult specimens. However, based on the data of a comparative morphological and morphometric study, it makes us think that it could be preliminarily identified as *G. soricis.*
- The genetic analysis of the larvae indicates that they could not be assigned to any of the different species for which data are available in the genetic database. The possibility that they could be a new species must be considered, so future exhaustive studies are necessary to analyses the morphological variability of adults, the morphology of the evolutionary stages, and the complete life cycles (hosts).

Much remains to be learned about the systematics of *Gongylonema*. Different studies (Makouloutou et al., 2013a, 2013b; Setsuda et al., 2016, 2018; Varcasia et al., 2017) have provided molecular data about *Gongylonema* genus, in line with our work. However, our data together with those of Esperón et al. (2013) indicate a possible greater diversity of *Gongylonema* species in Spain, and an evident scarcity of genetic information to support their genetic analyses. Future molecular analyses are needed for better understanding the ecobiology of *Gongylonema* in other regions and careful searching for more specimens in a European framework.

## References

[ref001] Alicata, J. 1935. “Early developmental stages of nematodes occurring in Swine”, Technical Bulletin 489 D. of Agriculture, Ed, Washington, DC.

[ref002] Casiraghi, M. , Anderson, T. J. C. , Bandi, C. , Bazzocchi, C. and Genchi, C. 2001. A phylogenetic analysis of filarial nematodes: Comparison with the phylogeny of *Wolbachia* endosymbionts. Parasitology 122:93–103. 11197770 10.1017/s0031182000007149

[ref003] Cordeiro, H. D. C. , Melo, F. T. D. V. , Giese, E. G. and Santos, J. N. Dos 2018. *Gongylonema* parasites of rodents: a key to species and new data on *Gongylonema neoplasticum* . Journal of Parasitology 104:51–59. 10.1645/17-3 29135391

[ref004] Cordero del Campillo, M. , CastaZón OrdóZez, L. and Reguera, Feo, A. 1994. Índice catálogo de zooparásitos ibéricos. Universidad de León, Secretariado de Publicaciones.

[ref005] Esperón, F. , Martín, M. P. , Lopes, F. , Orejas, P. , Carrero, L. , Muñoz, M. J. and Alonso, R. 2013. *Gongylonema* sp. infection in the scops owl (*Otus scops*). Parasitology International 62:502–504. 23872068 10.1016/j.parint.2013.07.005

[ref006] Esteban, J. , Fuentes, M. , Munoz-Antoli, C. , Saez, S. and Trelis, M. 2004. First report of *Gongylonema pithyusensis* (Nematoda: Gongylonematidae) in continental Europe. Helminthologia 41:173.

[ref007] Fernandez-De-Mera, I. G. , Gortazar, C. , Vicente, J. , Höfle, U. and Fierro, Y. 2003. Wild boar helminths: Risks in animal translocations. Veterinary Parasitology 115:335–341. 12944047 10.1016/s0304-4017(03)00211-5

[ref008] Ferrer Màrius Vicent, F. , Teresa Galan-Puchades, M. , Fuentes, M. , Cerezuela, A. and Galan, M. 2000. A helminthological survey of small mammals (Insectivores and Rodents) in the Serra Calderona mountains (Valencian Community, Spain). Research and Reviews in Parasitology 60:25–35.

[ref009] Hernandez-Rodriguez, S. and Gutiérrez-Palomino, P. N. 1992. *Gongylonema* (*Progongylonema*) *pacoi* n. subgen. n. sp. (Spiruroidea: Gongylonematidae) parasite D’oiseaux Corvidae. Annales de Parasitologie Humaine et Comparee 67:188–193.

[ref010] Kinsella, J. M. , Del Rosario Robles, M. and Preisser, W. C. 2016. A review of *Gongylonema* spp. (Nematoda: Gongylonematidae) in North American rodents with description of a new species from the cotton rat, *Sigmodon hispidus* (Mammalia: Cricetidae). Zootaxa 4107:277–284. 27394819 10.11646/zootaxa.4107.2.9

[ref011] Kumar, S. , Stecher, G. , Li, M. , Knyaz, C. and Tamura, K. 2018. MEGA X: Molecular evolutionary genetics analysis across computing platforms. Molecular Biology and Evolution 35:1547–1549. 29722887 10.1093/molbev/msy096 PMC5967553

[ref012] Lobo, J. M. , Martin-Piera, F. and Veiga, C. M. 1988. Las trampas pitfall con cebo, sus posibilidades en el estudio de las comunidades coprofagas de Scarabaeoidea (Col.) I. Revue d’Ecologie et de Biologie du Sol 25:77–100.

[ref013] Löbl, I. and Smetana, A. 2016. Catalogue of Palaearctic Coleoptera Series. Vol. 3: Scarabaeoidea, Scirtoidea, Dascilloidea, Buprestoidea and Byrrhoidea (Brill Academic Publishers, Leiden.

[ref014] Makouloutou, P. , Rana, H. B. , Adhikari, B. , Devkota, B. , Dhakal, I. P. and Sato, H. 2013b. A distinct genetic population of *Gongylonema pulchrum* from water buffaloes in Nepal. Journal of Parasitology 99:669–676. 10.1645/12-143.1 23421498

[ref015] Makouloutou, P. , Setsuda, A. , Yokoyama, M. , Tsuji, T. , Saita, E. , Torii, H. and Sato, H. 2013a. Genetic variation of *Gongylonema pulchrum* from wild animals and cattle in Japan based on ribosomal RNA and mitochondrial cytochrome c oxidase subunit I genes. Journal of Helminthology 87:326–335. 22967753 10.1017/S0022149X12000442

[ref016] Martin-Piera, F. and Lobo, J. M. 1996. A comparative discussion of trophic preferences in dung beetle communities. Miscellania Zoologica 19:13–31.

[ref017] Martín-Piera, F. and López-Colón, J. I. 2000. “Coleoptera, Scarabaeoidea I. En: Fauna Ibérica”, In Ramos, M. A. et al. (Eds), vol. 14, Museo Nacional de Ciencias Naturales. CSIC, Madrid, 526 pp.

[ref018] Mas Coma, S. 1977. *Gongylonema pithyusensis* N. Sp. (Nematoda: Spiruridae), Parasite Oesophagien du Lerot *Eliomys Quercinus* Ophiusae Thomas, 1925 (Rodentia: Gliridae) a Formentera (Baleares). Annales de Parasitologie Humaine et Comparee 52:13–18. 561564 10.1051/parasite/1977521013

[ref019] Milazzo, C. , De Bellocq, J. G. , Cagnin, M. , Casanova, J. C. , Di Bella, C. , Feliu, C. and Santalla, F. 2003. Helminths and ectoparasites of *Rattus rattus* and *Mus musculus* from Sicily, Italy. Comparative Parasitology 70:199–204.

[ref020] Mowlavi, G. , Mikaeili, E. , Mobedi, I. , Kia, E. , Masoomi, L. and Vatandoost, H. 2009. A Survey of dung beetles infected with larval nematodes with particular note on *Copris lunaris* beetles as a vector for *Gongylonema* sp. in Iran. The Korean Journal of Parasitology 47:13. 19290086 10.3347/kjp.2009.47.1.13 PMC2655331

[ref021] Mukaratirwa, S. , Pillay, E. and Munsammy, K. 2010. Experimental infection of selected arthropods with spirurid nematodes *Spirocerca lupi* Railliet & Henry, 1911 and *Gongylonema ingluvicola* Molin, 1857. Journal of Helminthology 84:369–374. 20132587 10.1017/S0022149X10000039

[ref022] Nichols, E. , Alarcón, V. , Forgie, S. , Gomez-Puerta, L. A. and Jones, M. S. 2017. Coprophagous insects and the ecology of infectious diseases of wildlife. ILAR Journal 58:336–342. 29036417 10.1093/ilar/ilx022

[ref023] Nichols, E. and Gómez, A. 2014. Dung beetles and fecal helminth transmission: Patterns, mechanisms and questions. Parasitology 141:614–623. 24476794 10.1017/S0031182013002011

[ref024] Nichols, E. , Spector, S. , Louzada, J. , Larsen, T. , Amezquita, S. and Favila, M. E. 2008. Ecological functions and ecosystem services provided by Scarabaeinae dung beetles. Biological Conservation 141:1461–1474.

[ref025] Poinar, G. O. 1975. Entomogenous nematodes: A manual and host list of insect-nematode associations. Leiden: Brill Archive.

[ref026] Portoles, E. , Granel, P. and Esteban, J. G. 1996. “Helminthofaunistic analysis of *Crocidura russula* (Hermann, 1780) (Insectivora: Soricidae) From the Albufera Natural Park (Valencia, Spain)”, Research and Reviews in Parasitology 56:203–211.

[ref027] Quentin, J. C. and Gunn, T. 1981. Morphologie et biologie larvaires de *Gongylonema soricis* Fain, 1955. Annales de Parasitologie Humaine et Comparée 56:167–172. 7196198

[ref028] Setsuda, A. , Da, N. , Hasegawa, H. , Behnke, J. M. , Rana, H. B. , Dhakal, I. P. and Sato, H. 2016. Intraspecific and interspecific genetic variation of *Gongylonema pulchrum* and two rodent *Gongylonema* spp. (*G. aegypti* and *G. neoplasticum*), with the proposal of *G. nepalensis* n. sp. for the isolate in water buffaloes from Nepal. Parasitology Research 115:787–795. 26531300 10.1007/s00436-015-4806-3

[ref029] Setsuda, A. , Ribas, A. , Chaisiri, K. , Morand, S. , Chou, M. , Malbas, F. , Muchammad, Y. and Sato, H. 2018. Molecular genetic diversity of *Gongylonema neoplasticum* from rodents in Southeast Asia. Systematic Parasitology 95:235–247. 29446034 10.1007/s11230-018-9778-0

[ref030] Suchard, M. A. , Lemey, P. , Baele, G. , Ayres, D. L. , Drummond, A. J. and Rambaut, A. 2018. Bayesian phylogenetic and phylodynamic data integration using BEAST 1.10. Virus Evolution 4:1–5. 10.1093/ve/vey016 PMC6007674 29942656

[ref031] Teichert, M. 1955. Biologie und Brutfürsorgemassnahmen von Geotrupes mutator Marsh. und Geotrupes stercorarius L. (Col. Scarab.). Wissenschaftliche Zeitschrift Martin Luther-University. Halle-Wittenburg 5:187–218.

[ref032] Varcasia, A. , Scala, A. , Zidda, A. , Cabras, P. A. , Gaglio, G. , Tamponi, C. and Sato, H. 2017. First record of *Gongylonema nepalensis* in domestic and wild ruminants in Europe. Veterinary Parasitology 246:11–18. 28969772 10.1016/j.vetpar.2017.08.022

[ref033] Xiaodan, L. , Zhensheng, W. , Ying, H. , Hongwei, L. , Jianqiu, J. , Peiru, Z. , Sha, S. and Zhimin, Y. 2018. *Gongylonema pulchrum* infection in the human oral cavity: A case report and literature review. Oral Surgery, Oral Medicine, Oral Pathology and Oral Radiology 125:e49–e53. 10.1016/j.oooo.2017.11.019 29329982

